# Vision Based Wall Following Framework: A Case Study With HSR Robot for Cleaning Application

**DOI:** 10.3390/s20113298

**Published:** 2020-06-10

**Authors:** Tey Wee Teng, Prabakaran Veerajagadheswar, Balakrishnan Ramalingam, Jia Yin, Rajesh Elara Mohan, Braulio Félix Gómez

**Affiliations:** Engineering Product Development Pillar, Singapore University of Technology and Design (SUTD); Singapore 487372, Singapore; weeteng_tey@sutd.edu.sg (T.W.T.); balakrishnan@sutd.edu.sg (B.R.); yin_jia@mymail.sutd.edu.sg (J.Y.); rajeshelara@sutd.edu.sg (R.E.M.); brauliofelixgomez@gmail.com (B.F.G.)

**Keywords:** wall following, CNN, HSR robot: wall cleaning, FCN8, SSD MobileNet, visual servoing

## Abstract

Periodic cleaning of all frequently touched social areas such as walls, doors, locks, handles, windows has become the first line of defense against all infectious diseases. Among those, cleaning of large wall areas manually is always tedious, time-consuming, and astounding task. Although numerous cleaning companies are interested in deploying robotic cleaning solutions, they are mostly not addressing wall cleaning. To this end, we are proposing a new vision-based wall following framework that acts as an add-on for any professional robotic platform to perform wall cleaning. The proposed framework uses Deep Learning (DL) framework to visually detect, classify, and segment the wall/floor surface and instructs the robot to wall follow to execute the cleaning task. Also, we summarized the system architecture of Toyota Human Support Robot (HSR), which has been used as our testing platform. We evaluated the performance of the proposed framework on HSR robot under various defined scenarios. Our experimental results indicate that the proposed framework could successfully classify and segment the wall/floor surface and also detect the obstacle on wall and floor with high detection accuracy and demonstrates a robust behavior of wall following.

## 1. Introduction

Cleaning of high-touch areas in a physical environment is very critical in preventing the spread of infectious diseases. One of the recent surveys on COVID-19 spread shows that the common mode of infection spread is by touching infected surfaces [1]. The frequently touched areas that include walls, doors, locks, handles, windows need to be cleaned within a fixed time interval. Among those, cleaning of large wall surfaces manually is always seen as a tedious, time consuming, and astounding task. Also, during the period of spread, there is a high possibility and risk where a human cleaner may get infected. To overcome such bottlenecks, and to reduce the risk of infection transmission, the cleaning contractors are encouraged to use robotic solutions by every developed nation. The use of robotic cleaners for professional cleaning services has been increased gradually in the past few years. Recent research done by the robotic industrial association states that more than 3000 industrial cleaning robots will be sold between 2019 and 2021. Successful market players include avid bot, eco bot, lions bot, Adlatus robotics and so on. Even though there are numerous market available robotic platforms which are considered as a replacement for the professional cleaners, most of these systems are concentrated only in floor cleaning, and not considered the wall cleaning. In the past two decades, numerous wall cleaning robots were researched with the wall climbing capability.

For instance, robots that climb vertical surfaces by vacuum-suction to a surface were created for the very purpose of wall cleaning. These adhesive robots are, however, known to carry the risk of it falling off due to several factors, one being, moving on a wall which causes air leakages due to its unevenness. A prototype was also made using dry-adhesives to allow the robot to scurry up walls in a lizard-like fashion [2]. Due to its lightweight, this robot is about to move quickly up walls with close to no resistance. However, it is unable to carry a payload heavier than itself [3]. Another example of a wall-climbing robot was also invented which is able to respond to various adhesion statuses of the wall and ensure the robot applies the right amount of suction to prevent it from falling [4]. However, with solely suction and lack of foundation built from the ground up, these wall-climbing robots will always pose a threat of falling and injuring passersby and also the environment by damaging buildings, floors, or statues. Rope climbing robots have also been invented for an additional level of safety [5]. However, not all walls have hooks in which a robot can support itself on. Also, most such climbing based wall cleaning robots concentrate on facades and often require human support to transfer from one wall to another. One alternate solution that was researched deeply in the past decades is using mobile based robots with wall cleaning mechanisms.

When we use a mobile robot to clean a wall, it is critical to detect and follow the wall. So far, numerous techniques have been used to detect the wall and follow it. For instance, robots using laser sensors have been used to ensure the robots stay on its path and are aligned to its path plan [5], such  methods allow the robot to make real-time decisions when it comes to clearing obstacles and avoiding running into people. Sensors on the wall following robots have proven to be an easy way of ensuring it follows its projected trajectory while avoiding obstacles. However, it is also known that sensors have a limited view of their path in which they have to follow [6]. This creates a problem when it is implemented in robots as obstacles could come from any direction and it is important that the system is able to detect this. To overcome this problem, another robot was invented with 24 sensor inputs, ensuring all possible views were covered and given to the robotic system [7]. These sensors were implemented on the robot’s waist and will only send a signal when it is close to a wall. Due to the height in which these sensors are placed, the robot will not consider hanging walls with no base (or receded base) and will move away from them, deviating from its purpose. However, robots that implement the use of sonar technology to aid in wall-following also exist [8]. Thus, despite its promising use, sonar technology has a lot of noise generated when receiving information back into the robot and would make filtering what is an obstacle or wall difficult to execute. Therefore, to effectively follow walls, the robot needs to see it from where it starts on the ground to where it ends before the ceiling, with as little difficulty and post-processing as possible. The robot also has to ensure it can avoid obstacles in real-time, further preventing any injuries from occurring during its operation.

Computer vision fused with Machine Learning (ML) and Deep Learning (DL) has become one of the emerging techniques. It has been widely used in image classification, image segmentation, object detection and tracking. It is also used in autonomous vehicles and mobile robot vision system for segmentation and recognition of objects in images. In the DL framework, Convolutions Neural Network (CNN) is the critical component for recognizing and locating the object in the captured image. In [9] CNN and Deep Belief Network (DBN) are combined for autonomous vehicle obstacle detection systems, where CNN is used for obtaining local information of objects and DBN is used for obtaining the global information of images. The combined architecture trained using KITTI data-set got an accuracy of 91.46%. Hua et.al developed an obstacle avoidance system for an outdoor robot. The authors used the dual-stage RGB-D semantic segmentation network ’RedNet’ and morphological processing algorithm in the robot vision system to detect the obstacles [10]. The dual-stage obstacle detection framework obtained 96.3% obstacle detection rate for indoor and 93.8% for outdoor. Another work has reported CNN based obstacle detection system in a complex environment. A four-layer CNN was used for feature extraction and SVM for classification. The network was trained with 10,000 images and detected the obstacles in the street with 83% accuracy [11]. Kore and Suchitra [12] proposed the real-time obstacle detection on-road using CNN, Local Binary Pattern (LBP), Local Ternary Pattern (LTP) and Gabor filter. Here, LBB, LTP and Gabor filter are used for feature extraction and CNN for obstacle detection. The methodology have a detection rate of 86.73% with 4% false rate. Bi-directional feature pyramid based hybrid CNN network was proposed by Li et al. [13] for deploying the obstacle avoidance system in unmanned surface vehicles. The Hybrid CNN network was built by combining the ResNet and DenseNet CNN framework and gained 93.80 mean Average Precision (mAP) for testing with the USVD2018 data-set.

Besides, DL algorithms are widely used in cleaning robots for trash detection, path planning and ARM manipulation, etc. In [14] author uses the deep learning framework in cleaning robot for recognizing and localizing the trashes from the robot vision module, where the author used deep CNN based semantic segment framework SegNet for segmenting out the ground region from other areas and ResNet for object detection and localization. The author reported that the combined approach obtained 96% trash detection accuracy and took 10.3 ms for ground segmentation and 8.1 ms for trash recognition. DL based object detection algorithms include SSD MobileNet, Faster RcNN ResNet and Yolo v2 are used to evaluate the trash and stain detection for cleaning robot application [15]. The author report that SSD MobileNet is an optimal algorithm for cleaning robot trash and stain detection, which provides a good balance between accuracy and computation cost. A deep learning architecture based autonomous cleaning robot was developed with learning ability to generate 2D trajectories from a human kinesthetic samples Jia Yin et al. [16,17]. Ref. [18] proposed a deep-learning framework for table cleaning tasks using Human support Robot. The authors used a 16 layer CNN framework build on the darknet framework. The developed model detects the food stain and food trash on the table with 95% detection accuracy. Taking account of the above facts, in this paper, we are proposing a novel vision-based wall cleaning framework that acts as an add-on for any professional robotic platform to perform wall only cleaning

The proposed framework is constructed with two deep learning framework to visually detect and segment the Wall/floor area and detect the floor obstacle and wall objects usually embedded on the surface such as doors, pipes, racks, switchboards, fire extinguishers and so on. Once the features on the walls are extracted, the system process the feature using a visual servoing technique to execute the robot wall following. The major challenges encountered during the development of the proposed scheme were its training of large data set, the integration of visual servoing techniques, autonomous operation and translating the theoretical design into a physical Human Support Robot (HSR) robot system. All these aspects are detailed in this paper, and concluded with the experimental results that validate the proposed scheme and its ability to wall follow with the HSR robot under three different scenarios. The proposed scheme is first of its kind that can perform visual based wall only following. The proposed scheme is an initial design towards the autonomous wall-following technique that can perform multiple tasks on a wall surface such as cleaning, painting, crack inspection, and so on.

This paper is organized as follows: Introduction, motivation, and literature review in Section 1, Section 2 discusses the proposed system, and Section 3 discusses HSR configuration. Experimental results are detailed in Section 4. Finally, Section 5 concludes this research work.

## 2. Vision Based Wall Following and Obstacle Avoidance Framework

**Figure 1:**
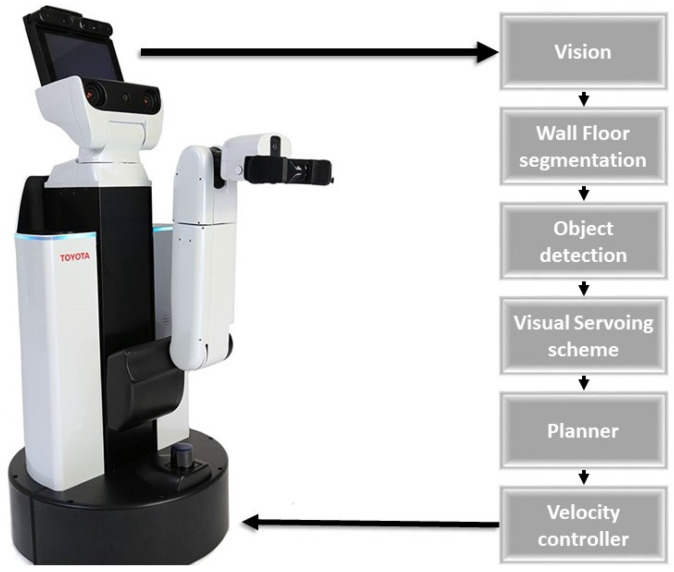
Overview of the Proposed Scheme.

The main objective of our proposed system is to use a mobile robot to autonomously follow the wall for cleaning the wall surfaces that have been frequently touched. Figure 1 shows the flow diagram of the proposed system that enables the wall following ability of any mobile robot. In this paper, we utilized Toyota Human Support Robot as a case study to evaluate the scheme. Algorithm 1 shows the execution flow of the wall following and obstacle avoidance system. The first step of our proposed method is to segment the wall and floor surface from the image that was captured from the front camera of the robot by utilizing the semantic segmentation CNN model, FCN8 [19]. Once the segmentation is completed, we separate the wall surface and floor surface by applying the mask on the segmented wall and floor region on the segmented image and passed it to the next stage for further processing. In every wall and floor surface, there are few objects that need to be avoided that include obstacles on nearby wall and objects fixed in the wall include door, switch boxes, pillars, fire extinguishers, sandal racks, and so on. To identify such objects, we adopt SSD MobileNet object detection framework [20,21,22] as a next step. These objects will be identified from the masked segmented wall and floor images, which is used as primary information by the system to decide whether to follow the wall or not. If there is no object detected nearby the wall, the visual servoing technique gets activated to generate the trajectory to follow the wall. The generated trajectory is then subscribed by the velocity controller to produce a motor primitive that executes the locomotion. The detail of each module is described as follows.
**Algorithm 1** Wall Following and Obstacle Avoidance 1:**Input:** 2:Image capture from HSR vision system 3:**Output:** 4:Alert signal (Alert_HSR) to HSR for obstacle avoidance and safe cleaning 5:**Initialize:** 6:*C* is an Camera 7:*F* is an Image frame 8:Segment_array: hold the segmented image 9:Wall_Segment: hold the segmented wall image10:Floor_Segment: hold the segmented floor image11:Object_wall: hold the detected objects on wall12:Object_floor: hold the detected objects on floor13:**Initialize End**14:**Begin:**15:**while** (1) **do**16: *F* = Capture Frame (C)17: Segment_array = FCN8_ segmentation(*F*)18: Wall_Segment = Segment_array && floor_mask    ; mask the floor19: Floor_Segment = Segment_array && wall_mask    ; mask the wall20: Object_wall = SSD_MobileNet (wall_segment)    ; Object detection on segmented wall region21: Object_floor = SSD_MobileNet (floor_segment)    ; Object detection on segmented floor region22: Distance_floor-obstacle_wall ( wall_segment, floor_segment)    ; compute the distance of the detected floor object from wall23: Wall_Object-distance_ground( wall_segment, floor_segment)    ; compute the distance of the detected wall object from ground24: **if** (Distance_floor−obstacle_wall) **then**25:  Alert_HSR( obstacle near by wall)    ; Alert to HSR to avoid obstacle on path26: **end if**27: **if** (Wall_Object−distance_ground ) **then**28:  Alert_HSR( object on wall)    ; Alert to HSR for cleaning modules safe operation29:  goto $\overset{}{\to }$ Begin30: **else**31:  goto $\overset{}{\to }$ Begin32: **end if**33:**end while**

### 2.1. Fully Convolutional Network for Wall Segmentation

**Figure 2:**
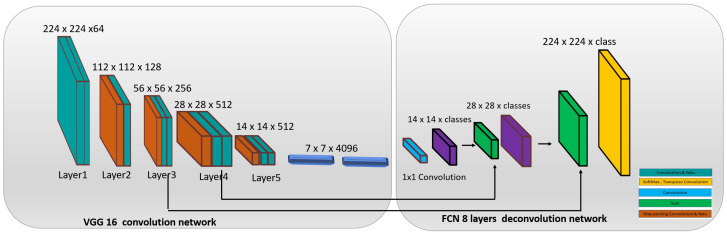
FCN8 Network architecture.

In this work, FCN8 is used for segmenting out the wall and floor region from captured image [19,23]. It is the first semantic segmentation based CNN framework developed by Shelhamer et al. [19]. Figure 2 shows the internal architecture of FCN8. It comprises three key components including convolution network and deconvolution network and SoftMax probability function for finding the pixel class. FCN8 adopts VGG16 architecture for the convolution network, which contains 15 convolution layers, five 2×2 max-pooling layer and stride function 2 between convolutions layers. In the convolution network, each pooling layer down-sample the input by a factor of 2 both horizontally and vertically. In the fifth pooling layer, the input image is downscaled by a factor of 32. The deconvolution network is placed after the last fully connected layer of the VGG16 network. The deconvolution layer performs the up-sampling task (make the output equal to the size of input image ) using unpooling operation. To overcome the spatial information loss by convolution network, layer 4 and 3 of VGG16 was connected with layer 9 and 10 of deconvolution network and upsampled for 2 times to match with dimensions of layer 4 and 3. Finally, for generating the full-resolution segmentation map similar to input image size, the FCN Layer-10 is upsampled 4 times in FCN layer 11 using following parameters 16 kernel, stride = (8,8) and paddding (’same’).

### 2.2. Obstacle Detection Framework

Obstacle detection is a key function in our proposed system. It helps the robot to avoid the obstacles nearby wall and also helps to prevent the cleaning module collision with objects fitted in the wall while performing the cleaning task. To perform obstacle and object detection, a deep-learning based object detection framework is adopted in this work. Generally, an object detection framework is two types, one-shot detector and two-shot detector. The one-shot detector is quite fast contrast with two-shot approach algorithms such as Regional proposal networks (RPN). The single-shot algorithm requires one shot to detect multiple objects inside the image, while the RPN series (Fast RCNN, Faster RCNN) required two shots, one for producing the region proposals and another for detecting the object from generated proposals. Moreover, SSD is a lightweight framework widely used in mobile robot applications and also run in low power embedded devices. Hence we adopt the SSD framework for the obstacle detection task. In this work, SSD is combined with MobilNet feature extractor instead of VGG16 feature extractor to detect the obstacle and objects in real-time.

**Figure 3:**
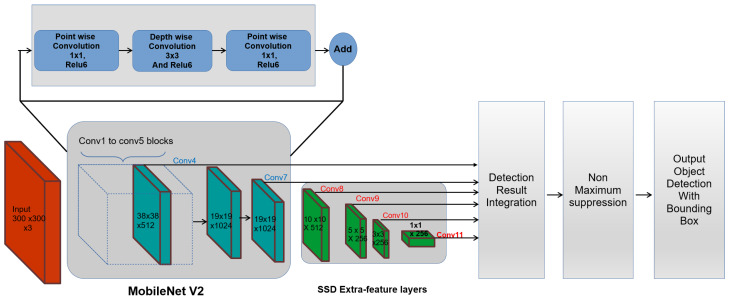
SSD MobileNet functional block diagram.

The Figure 3 shows an overview of the SSD MobileNet object detection framework. Here, SSD is run on top of the MobileNet framework and performs the object localization task, which uses the MobileNet as a feature extractor. In SSD, VGG-16 is a default feature extractor and classification network. However, in this work, VGG-16 is replaced by the MobileNet feature extractor, which is introduced by google. MobileNet is an optimized deep neural network architecture that will run very efficiently on mobile devices and low computing CPU.

In MobileNet framework, conventional convolutional layers are modified by depthwise separable convolution layers and pointwise convolution function. Here, the depth wise functions perform deep convolutional operations using 3×3 kernels and pointwise convolutional are common convolutional layers that use the 1×1 kernels. Further batch normalization and ReLU 6 is applied to each convolutional result. The batch normalization functions fine-tune the data by setting the learning parameter, adjusting the learning rate, dropout ratio and restricts the gradient disappearance. The combination of deep wise and point wise convolutions structure activate the MobileNet to speed up the learning process and also reduce the computation time. Finally, the model has a residual connection, which gives the architecture a better accuracy compared to non-residual architectures.

**Figure 4:**
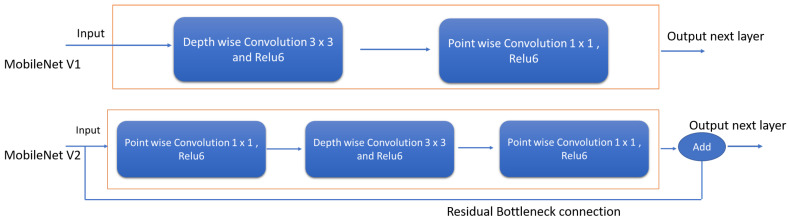
Difference between MobileNet V1 and MobileNet V2.

In our work, MobileNet v2 is adopted for feature extraction, which is an updated version on MobileNet V1. The Figure 4 shows the difference between the MobileNet V1 and MobileNet V2 architecture, where depthwise separable convolutional blocks and pointwise convolutional layers similar to V1. However, there is a slight change in the structure of the convolution layers. Here, the first layer is 1×1 convolution with ReLu6 and the second layer performs the depth-wise convolution operation and third layer does the 1×1 convolution without any non-linearity. Further, the residual layer structure of MobileNet v2 is modified based on the structure of ResNet architecture, which helps to improve the accuracy of depthwise convolution layer without having large overhead. Bottleneck layers that reduce input size are also used. An upper bound is applied to the ReLU layer, which limits the overall complexity.

To carry out the obstacle and object detection task, SSD replaces the final few fully connected layers in MobileNet with additional convolution layers to localize and detect objects using the feature maps. It includes the pyramid structured multiple auxiliary convolutional layers on top of MobileNet to extract the feature maps at different resolutions. In SSD, six output feature maps are used for detecting the class of an object and localizing the form of a bounding box where first two (19×19) are taken from MobileNet feature extractor and remaining feature maps (10×10,5×5,3×3,1×1) are generated by auxiliary convolution layers.

While training, the loss for each prediction is computed as a combination of the confidence loss $L_{confidence}$ and location loss $L_{location}$ (Equation (1)). The confidence loss is the error in the prediction of class and confidence. The location loss is the squared distance between the coordinates of the prediction. A parameter α is used to balance the two losses and their influence on the overall loss. This loss is optimized through the use of the Root Mean Squared gradient descent algorithm [24]. This algorithm computes the weights $w_{t}$ at any time *t* using the gradient of loss *L*, $g_{t}$ and gradient of the gradient $v_{t}$ (Equations (2)–(4)). Hyperparameter β,η are used to balance the terms used for momentum and gradient estimation, while ϵ is a small value close to zero for preventing divide by zero errors.

(1) $$L=\frac{1}{N}\left(L_{confidence}+\alpha L_{location}\right)$$

(2) $$v_{t}=\beta v_{t-1}+\left(1-\beta \right)g_{t}^{2}$$

(3) $$\Delta w=\frac{-\eta }{\sqrt{v_{t}+\epsilon }}\times g_{t}$$

(4) $$w_{t+1}=w_{t}+\Delta w$$

### 2.3. Visual Servoing

**Figure 5:**
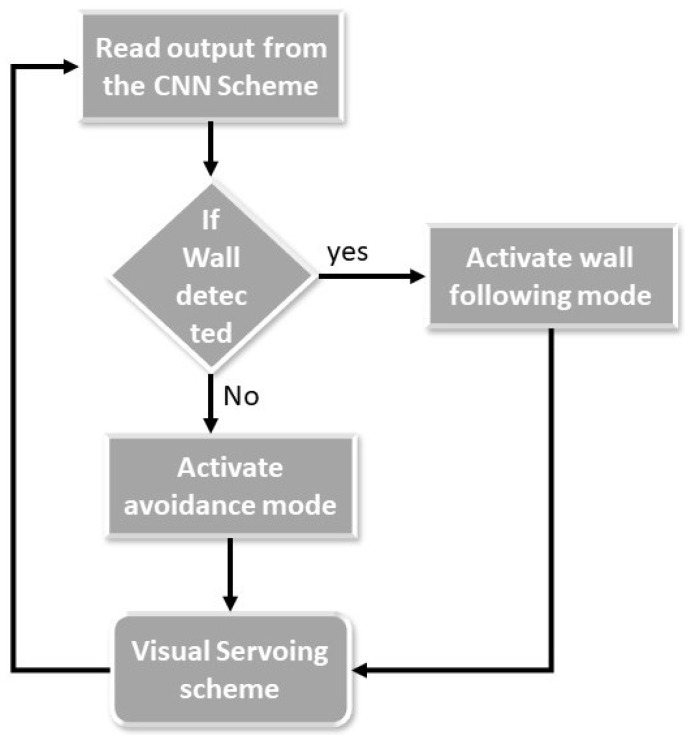
Operational Flow Diagram.

The main objective of the visual servo scheme is to design a velocity controller for a robotic system that minimizes the error *e* generated between a chosen visual features *s* and its desired values $s^{*}$ in the image frames [25]. Since our aim is to design a control function that could curtail the error value $e={\left[s-s^{*}\right]}^{T}$, the features need to be selected that can be visually traceable by the vision system. Also, it is critical to choose the right feature with respect to the application. In our case, we are following the wall with a particular distance in order to clean the wall surface. So, the first feature that we chose was $\theta_{rm}$ made by the *z*-axis of the robot’s vision system with respect to the wall/floor separation line of the corridor. This feature will be zero when the robot is looking forward and the camera is parallel to the wall surface. The *x*-coordinate $x_{ob}$ of the center point in the detected object $ob_{t}=\left(x_{ob},y_{ob}\right)$ at the time instant *t* is chosen as second feature. Both the features could be extracted from the output images of the CNN network. Figure 5 illustrates the operational flow of our wall following algorithm. The algorithm initially checks the non wall features in the output images from the CNN. According to the findings, the algorithm decides whether to follow the wall or to avoid them.

**Figure 6:**
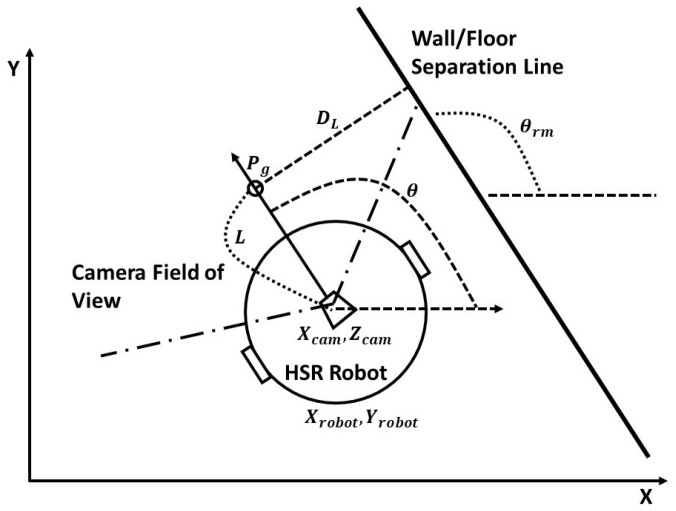
HSR robot navigates along a wall/floor segmentation line.

During the wall following operation, the system first took the feature information and navigates in consonance with the relative position and orientation with respect to the separation line. While navigating, the path has been generated up-to the goal point $P_{g}$ which is calculated from the constant look up distance *L* from the center of the camera. For every new frame, the $P_{g}$ will be increased for the *L* distance according to the current position of the robot. The relative position is calculated from the robot starting point and the off-set distance $D_{L}$ which will be used as a gap that needs to be maintained from the wall. The relative orientation is calculated as deviation from the detected feature $\theta_{rm}$ i.e., $\theta -\theta_{rm}$, shown in Figure 6. Based on the kinematic model of the desired robot and with the shown model, we can able to generate the velocity commands for the robot.

**Figure 7:**
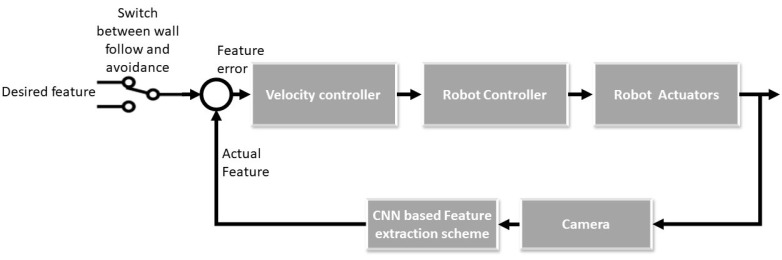
Feedback control loop for wall following.

As mentioned earlier, the key distance value that our system needs to maintain to achieve the wall following operation is $D_{L}$. These values will always be constant unless there is an obstacle detected in the frame. When the obstacle is detected, the $D_{L}$ will be increased and the value will be calculated not from the wall separation point but from the center point of the second detected feature $x_{ob}$. According to the selected feature, the feature error was generated, which aids in producing the velocity commands in the velocity controller. The velocity values are passed to the robot controller unit which converts the commands to appropriate motor RPM with reference to the robot’s kinematics. The robots wall following motions are generated which will update the image frames and its features. The feature errors are again calculated and the same process is continued till the robot completes the task. The feedback loop of the proposed system is shown in the Figure 7.

## 3. HSR Robot

**Figure 8:**
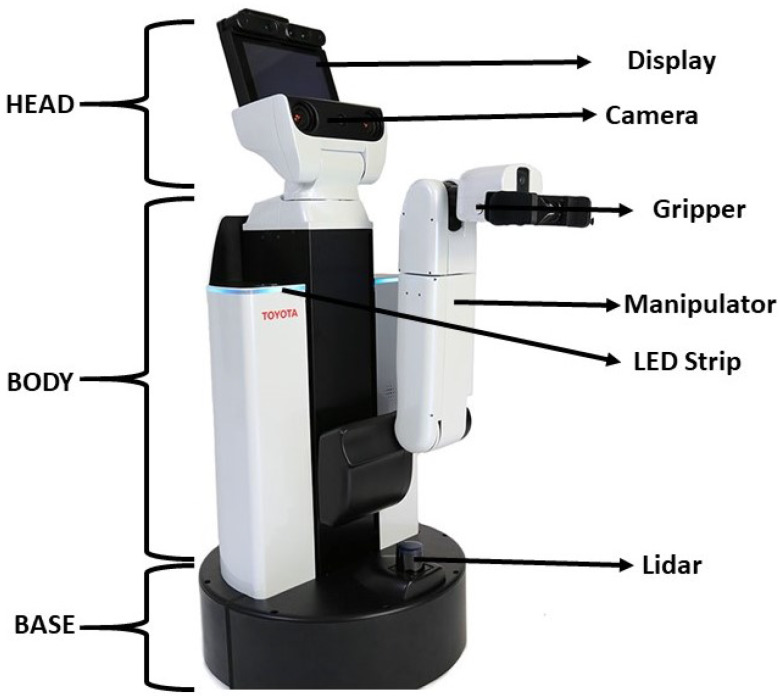
HSR robot System.

**Figure 9:**
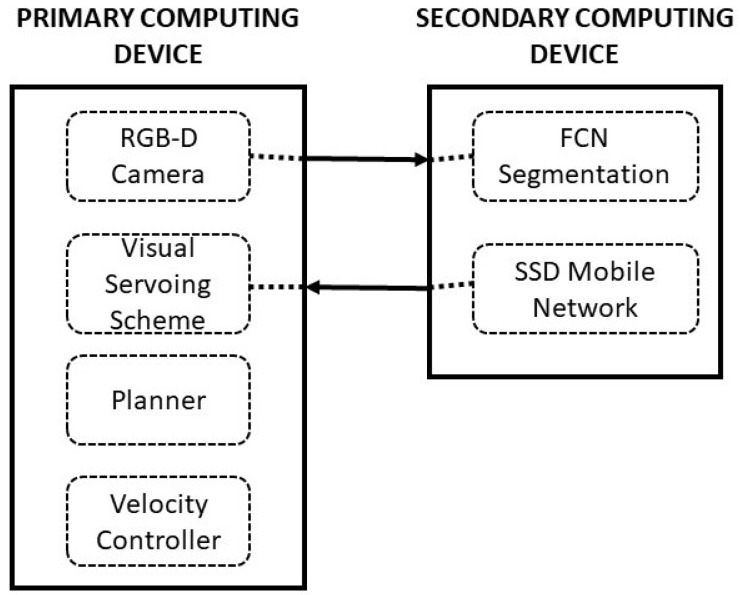
HSR computing architecture.

In this article, we utilized Toyota HSR as our experimental platform to validate the proposed system. This section describes the HSR robotic architecture which provides an overall view of the implementation of the proposed scheme, autonomous navigation, and the perceptional components that aids in system execution. HSR is a mobile manipulator platform developed by Toyota which consists of a set of different sensor and actuator modules. The robot is equipped with an RGB-D camera (specification on Table 1) on top of its head with a display, manipulator with four degrees of freedom, mobile base module, and system body module which consist all computational equipment shown in Figure 8. The system runs with the Robot operating system (ROS). All operations of wall following runs under the ROS architecture. The ROS system enables the connection and linkage between the perceptional modules and the execution modules. All positional transformation of each module is pre-linked in the system which gives better localization values. The positional transformation all done in the 3D space wich will be used in our susyem to estimate the selcted feature. Figure 9 shows the schematic representation of HSR hardware architecture configuration, which comprises of two computing units, namely primary computing, and secondary computing. The first computing system is where we run our primary OS ubuntu within the ROS system will be functioning. This computing system takes the authority to communicate between each controllers, drivers that runs sensors and actuators. Also, this computing device is taking the lead manages all types of communication between the user. The display of HSR is connected with the primary computing system internally to monitor the process.

The second computing system is meant to take care of all vision system processes. Nvidia’s Jetson TK1 board (GPU) is used in the secondary computing unit to execute the vision system task. This system acts as a slave for the primary computing device, so ROS also consider this system as a slave device. The primary function of this system is to compute all the received images and convert the information into ROS message format and sends back to the master system. Also, this system is responsible for receiving the ROS messages and decodes to pass it to the deep learning framework. Both the primary and the secondary systems are connected over Transmission Control Protocol/Internet Protocol (TCP/IP) and share all the ROS topics.

To execute the wall following task, the primary computing system activates the ROS connection and establishes the communication between each module. Once the connection is established, the ROS system takes over to execute the wall following function. The first step that the system performs is to activate the RGB-D camera and convert the video stream into separate image frames to pass it to the secondary computing for processing. The secondary system runs the FCN8 framework and SSD MobileNet framework to perform the segmentation process and object detected process respectively. The processed images are passed back to the ros layer as appropriate ros messages.

It is critical for the robot to properly localize itself for a robot to perform error-free navigation. The HSR is built with various sensors that can be used as and fused to perform localization. In our case, we fused various sensors information such as 2D lidar, camera, wheel encoder, and the IMU to identify the current location. We used traditional SLAM [26,27], and localisation [28] techniques to perform the localisation. We utilized preexisting ROS packages to run on the ROS networks. Once the robot is activated the ROS system starts running the localization module to get the global position of the robot. Eventually, the ROS layer sends the request to the secondary computing device to share the feature information. Once these two information’s are available, the system calculates the relative position as explained in the previous section. With respect to the field of view from the camera, the goal point has been defined which generates the path and velocity commands.

**Figure 10:**
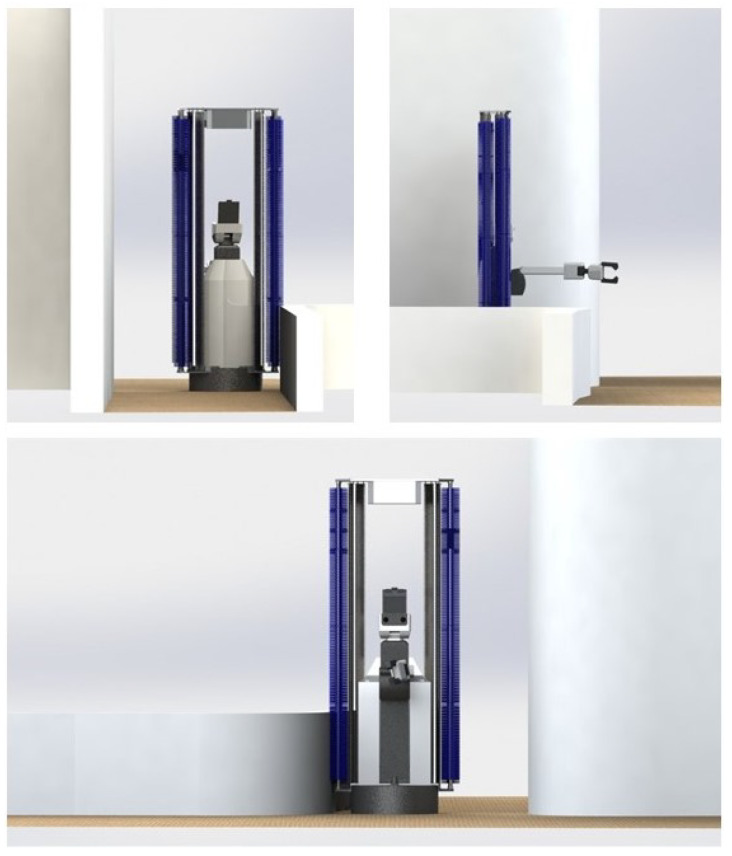
HSR Robot with cleaning payload.

The velocity commands are subscribed by the velocity controller which produces the motor primitives. The HSR motion is designed under the principle of differential drive, so the controller generates two main motor frequency $L_{pwm}$ and $R_{pwm}$. The HSR equipped with the 24 *V* DC motor that drives the robot. These motors are connected with the motor driver which is controlled by the arm controller that communicates with the robot’s primary computing system. Modification of the HSR robot needs to be carried out by attaching the cleaning payload on both sides to execute the wall cleaning task. The cleaning payload consists of a rotating mechanical brush that cleans high touch areas on the walls. The rotating mechanism works by securing a motor to the brush. When power is supplied, it will allow the brush to rotate. The CAD model of the modified HSR robot is shown in Figure 10. However, this paper focuses on the successful completion of the wall following without the cleaning payload. The Figure 10 is provided to give an overview idea of our proposed system’s end application.

## 4. Experimentation and Results

This section describes the experimental procedure and results obtained from the proposed system. The experiment was designed with three steps: data-set preparation and training the deep learning frameworks, evaluating the trained model in offline using indoor data-sets and testing with HSR robot platform. Here, the segmentation model performance was evaluated with pixel classification accuracy, Intersection Over Union (IoU) and F1 score metrics [19] and obstacle detection model was assessed through precision (Equation (5)), recall (Equation (6)) and F1 score metrics (Equation (7)).

(5) $$Precision\left(Prec\right)=\frac{tp}{tp+fp}$$

(6) $$Recall\left(Rec\right)=\frac{tp}{tp+fn}$$

(7) $$F_{measure}\left(F_{1}\right)=\frac{2\times precision\times recall}{precision+recall}$$

Here, tp,fp,tn,fn represents the true positives, false positives, true negatives and false negatives respectively as per the standard confusion matrix.

### 4.1. Data-Set Preparation and Training

**Figure 11:**
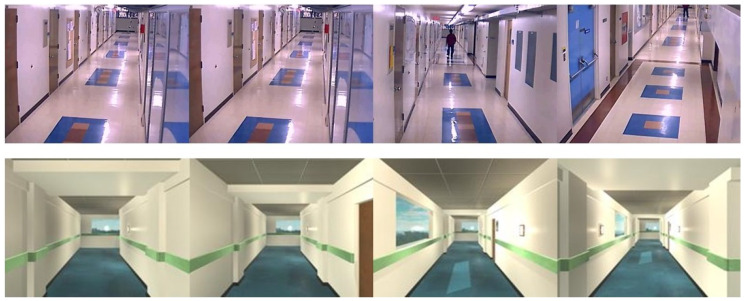
Training data set for wall/floor segmentation.

**Figure 12:**
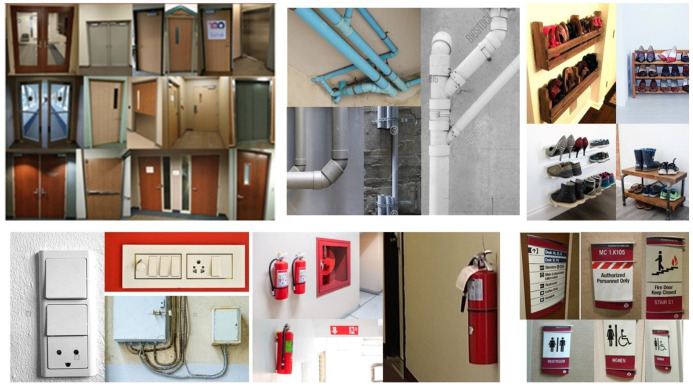
Training data set for object detection.

The data-set preparation process involves collecting indoor images with different obstacles and different wall and floor background. We collect the data-set from existing indoor image database through online [29,30,31,32,33,34,35,36] for both corridors and the obstacle images. However, some obstacles data is not clear and not from the perspective of the robot. So we used the robot camera to capture a few images of the obstacles from the perspective of HSR robot. The specification of the RGB-D camera is given Table 2. In total, there are 8000 images collected, which contains various types of obstacles that include fire extinguisher, photo frame, signboard, wall ornaments (wall clock, decorative lights), safety box, switch box, furniture (chair and rack), window, door, pipes, dustbin and plant pot. Some sample images were used for training the segmentation, and object detection network is shown in Figure 11, and Figure 12 respectively.

To improve the network learning rate and controlling the over-fitting, the data expansion methods are applied in collected images. Here, image scaling, rotation and flipping method are used to increase the number of sample images in the data-set. Further, in our experiment, the image size of (640×480) resolution was used for both training and testing. The collected image data set were separated into for FCN and the SSD Mobile Network. The corridor’s data sets were trained for the segmentation process in FCN. On the other hand, we used the obstacle image data set to train the SSD Mobile Network. The two CNN models (FCN and SSD MobileNet) was developed in Tensor-flow 1.9 Ubuntu 18.04 version and trained using following hardware configuration Intel core i7-8700k, 64 GB RAM and Nvidia GeForce GTX 1080 Ti Graphics Cards.

**Figure 13:**
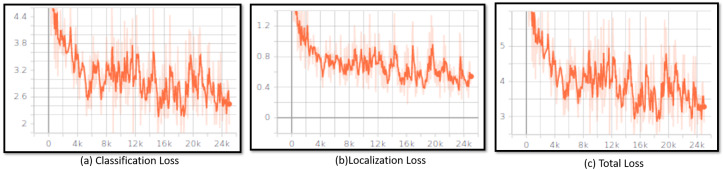
Training Results.

Figure 13 shows the results obtained from the training stage for the object detection model. The training was run 25,000 iterations and tracks the loss functions like classification loss, localization loss and total loss. In this process, we observe that loss was minimized each iteration. In the starting stage, the classification loss was above 4.4, the localization loss is above 1.2 and the total loss was above 8 after 25,000 iteration the total loss, classification loss and localization loss was gradually reduce and reach bellow 3.2, 2.4 and 0.6. The graph result shows that loss is decreasing over iteration and loss fluctuation is stable at 25,000 iterations, which indicate that the model converges and is trained properly.

#### Optimization

The two networks are trained with mini-batch SGD algorithm and network hyperparameters are obtained from a pre-trained model VGG16 [37] and SSD MobileNet trained by Ms COCO data-set [38]. The networks were fine-tuned with a batch size of 32 and trained for 10,000 epochs. The network uses the learning rate of 0.0002 and a momentum of 0.9, respectively. Further, a k-fold cross-validation procedure is used to evaluate the training image data-set. In this scheme, images are split into k groups and take k-1 for training the network, and the remaining one set is used for testing. In our work, we use 10 fold cross-validation scheme. The experimental results images shown are obtained from the model with good accuracy.

### 4.2. Offline Test with Indoor Data-Sets Images

**Figure 14:**
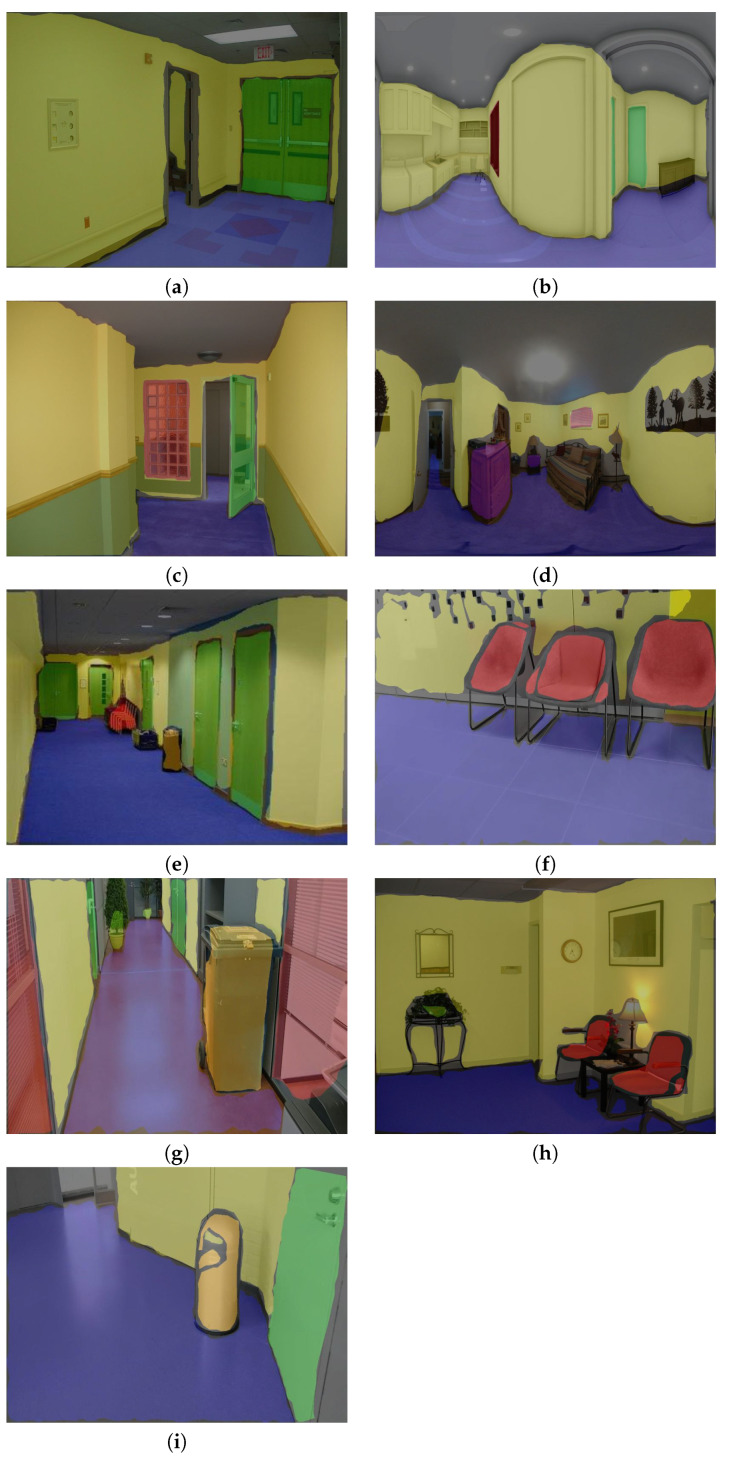
Offline experiment FCN8 segmentation result.

**Figure 15:**
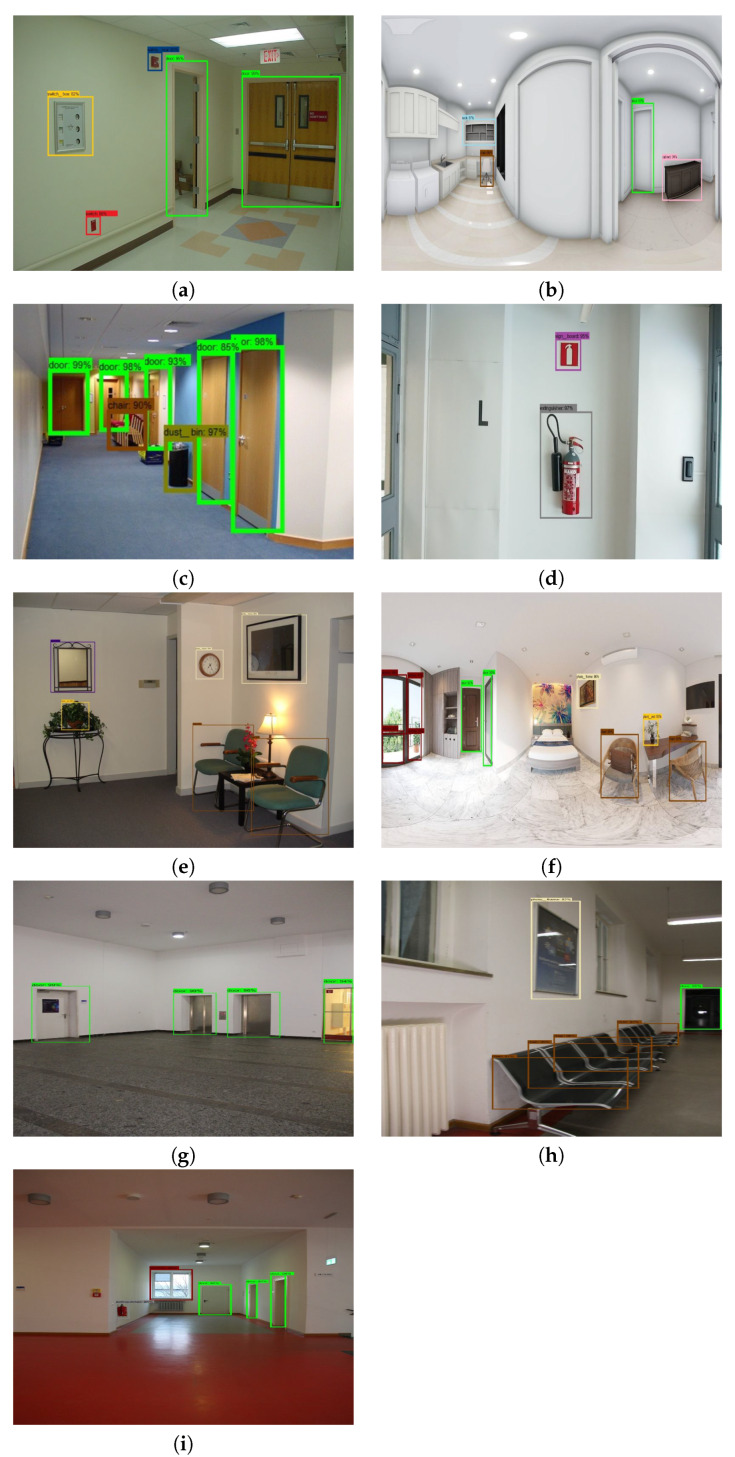
Offline experiment SSD MobileNet Obstacle detection result.

To carry out the offline test, the models inference graph is configured into HSR and tested with four indoor image database include NAVVIS indoor database [33], Quattoni et al. indoor scene database [34], shih et al. 360-indoor dataset [35] and Adhikari et al. indoor object detection dataset [36]. To verify the robustness of the system, the test images are chosen with different wall and floor colors and images with different lighting conditions. The object fitted in the wall and located nearby wall are only considered as an obstacle. There are 1200 images (consider each class 50 count) chosen for the offline experiment. The segmentation and detection results of the indoor image database are shown in Figure 14a–i and Figure 15a–i detection, and performance metrics results are reported in Table 2.

The experimental results indicate that the segmentation framework successfully differentiates the floor and wall from complex test images includes different lighting conditions and different wall and floor color and obtained pixel accuracy is above 90%, IoU average of 91.08% and average F1 score of 90.93% respectively. In this experiment, we observe that the fall of detection accuracy in some classes is due to false classification (ex: false classification between signboard and photo frame) and miss detection due to the tiny size of objects. ( Ex: safety box, switch box). However, this will not heavily affect the functionality of the robot. Further, the table result Table 2 indicate that the accuracy of SSD MobileNet frame for obstacle detection is above 90% and detects the obstacles on the wall and nearby wall with above 92% confident level. The offline experimental result proves that the proposed system is more suitable for deploying in HSR for real-time obstacle detection and avoidance.

### 4.3. Real-Time Test

**Figure 16:**
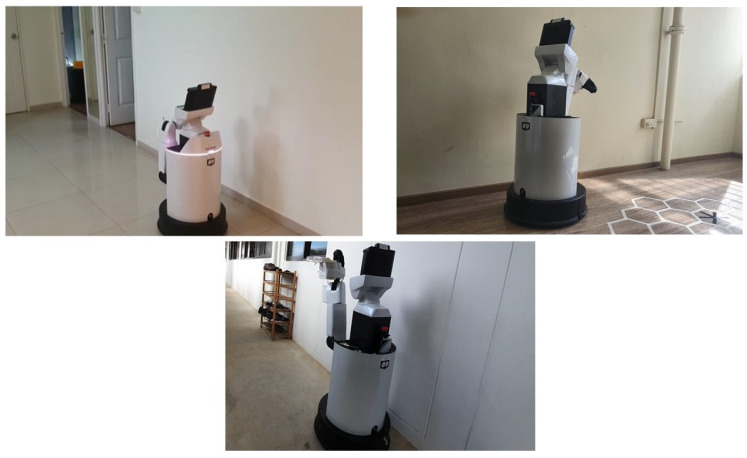
Experimental Scenario-1 (**top left**), Scenario-2 (**top right**), Scenario-3 (**bottom**).

The Real-time test was performed with HSR robot under various scenarios that have a straight corridor that exists with few obstacles along the wall surface. Before started the experiment, the robot was switched on and let the robot to reboot the internal system for few minutes. After the successful reboot of the robot, the robot will undergo a self-calibration mode, which calibrates the head position, and arm position to go back to its initial position. Then the robot was drove manually using the joystick controller to the testing spot. The user specify the wall direction (left or right wall) of which the robot is going to follow. Once the robot is positioned properly from the starting point, the autonomous wall following programming will be activated. The robot will start the wall following and avoidance process for a defined distance. The end distance will be varied according to the corridor chosen and its length. In the first scenario, we tested the robot on a straight wall with a length of 2 m shown in Figure 16(top left). In the second scenario, we chosen a straight wall with a distance of 1.7 m Figure 16(top right). In the final scenario, the corridor is chosen for a distance of 2.3 m Figure 16(bottom). In all considered scenarios, there are unique obstacles presented along with the wall surface. Our aim behind conducting this experiment is whether the robot could able to avoid those obstacles with the proposed scheme and only follows the wall. In the coming section, we have discussed on the results which we separated as neural network results and wall following results which is an outcome of visual servoing.

**Figure 17:**
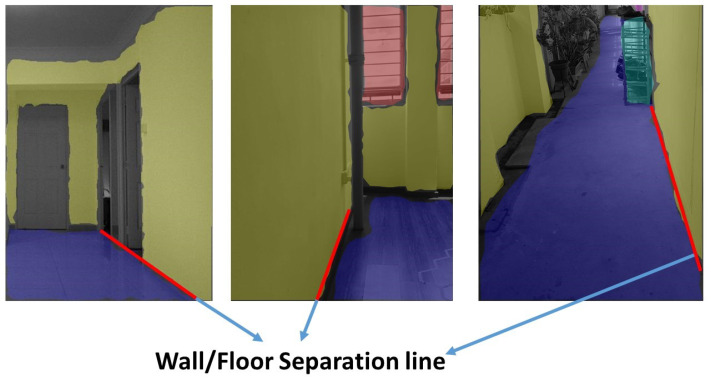
Wall/floor segmented result of scenario-1 (**left**), scenario-2 (**middle**), scenario-3 (**right**).

**Figure 18:**
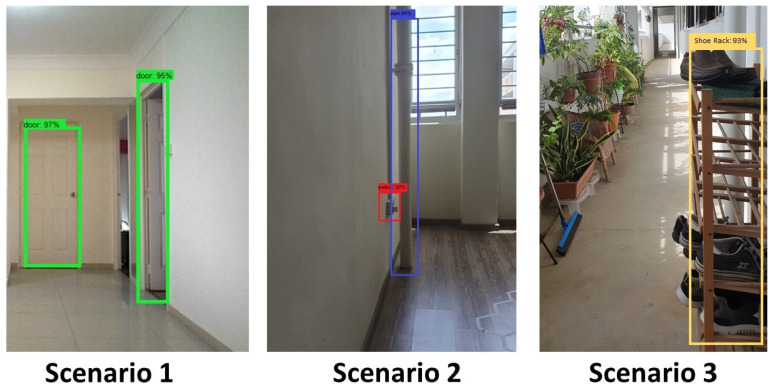
Object detection result during trials in scenario-1 (**left**), scenario-2 (**middle**), scenario-3 (**right**).

The experimental trials were initiated by manually driving the robot to test scenario 1 starting point. Then the autonomous wall following scheme was initiated. Once the program starts executing, the camera got activated to publish the image frames to the secondary computing device. The trained model of FCN8 that is running on the secondary system starts to segment the images as floor and wall. The Figure 17(left) shows some of the segmented images during the experiment-I trials. The result demonstrates the ability of the system to successfully segment the image frame as wall/floor and could identify the separation line. In this case, the wall/floor segmentation has obtained high pixel-wise accuracy of 89.2 %. Further, the output image is again processed for detects the obstacles that present along the wall surface. Figure 18(left) shows the output of the SSD mobileNet that shows the detected obstacle on the segmented wall surface, in a closed-door scenario. For such obstacle detection, the system has detected the obstacle with 95% confidence level.

In the second set of experiments, the robot is placed in the starting point of scenario 2. We followed the same procedure to perform the wall following task. In scenario 2, the illumination was quite dimmer than the previous scenario as you can see in the Figure 16(top right) However, total successful wall/floor segmented frames that the proposed system obtained 88.5% pixel-wise accuracy. Further, the obstacle detection algorithm detects the obstacle (PVC pipe) on a segmented region with 90% confidence level. Figure 17(middle) and Figure 18(middle) illustrates the outcome of the segmented images and the obstacle detection on the segmented wall surface respectively. Corresponding to the trial in scenario 3, the procedures were pursued as the previous trials. In this case, the obstacle that we considered was a shoe rack. In this trial, the illumination is much higher than the first scenario. Figure 17(right) depicts the results that were generated from the FCN. Throughout the trial, the total percentage of the number of frames that has been successfully segmented with 89.5% pixel-wise classification accuracy. Regarding the obstacle detection the Figure 18(right) shows the obstacle detected frame on the segmented wall surface. In this case study, the object detection algorithm detects the outdoor obstacle (shoe rack) with 93% confidence level. In this analysis, we observe that there are some miss classification in the segmentation process. This miss detection was encountered due to the similarities in the features information between floor and wall, sensor noise, and perspective viewpoint. We will consider using few depth data directly for the segmentation and identification process in the future works to reduce the noise and improve the accuracy.

#### 4.3.1. Performance Analysis

Table 3 and Table 4 summarise the performance metrics result for offline and real-time case study of segmentation and obstacle detection results.

The table results Table 3 indicate that the trained FCN model obtained an average of (offline and real-time) 89.47% pixel classification accuracy, 89.97% mean IoU, and 89.84% F1 score respectively. On the other-hand SSD MobileNet detects the obstacle on segmented image region with an average precision of 91.87%, recall, F1 score and accuracy of 90.77 and 91.01, 89.44 respectively. The experimental results show that the performance of segmentation and obstacle detection framework efficiency is stable for both offline and real-time test.

#### 4.3.2. Comparison Analysis of Different Architectures

The performance of the segmentation model and object detection framework was compared with other popular segmentation and object detection framework. In this analysis, FCN-AlexNet, FCN16 framework, was considered to compare the performance of the FCN8 segmentation framework. Similarly, Faster RCNN ResNet and Faster RCNN Inception and YOLO V2 frameworks are used for comparison with the SSD MobileNet object detection model. The models are trained using the same data-set and a similar amount of time and tested in NVIDIA GPU cards with 1200 test images used in the offline experiment. Table 5 and Table 6 shows the comparison analysis of different architecture.

The Table 5 results indicate that FCN8 obtained better pixel-wise classification accuracy than FCN AlexNet and FCN16. Furthermore, the segmentation accuracy of FCN AlexNet is very low for object class with small pixel areas. Further, object detection comparison results indicate that Faster RCNN ResNet and Faster RCNN Inception model have better performance than SSD MobileNet and Yolo framework. However, those model computation time is quite high. Those uses Regional Proposal Network (RPN) require two-shot to detect the multiple objects in an image (one for generating the region proposal and one for detecting the object). Hence its computation time is quite high compare to one-shot detection scheme SSD and Yolo, and hard to run in low power embedded platforms and mobile devices in real-time. YOLO based object detection models are faster compared to SSD and RCNN series, but detection accuracy is poor, specifically small object detection. SSD-MobileNet shows a good balance between accuracy and computation cost and its inference time is quite fast enough to be used in real-time object detection and obstacle detection task and specifically in mobile robot platform.

#### 4.3.3. Evaluation of Wall Following and Avoidance

**Figure 19:**
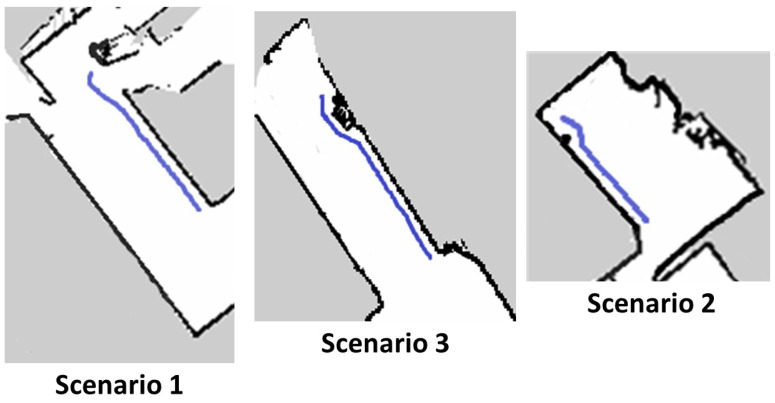
Generated path of HSR robot after completing the wall following task in scenario 1 (**top left**), scenario 2 (**top right**), scenario 3 (**bottom**).

**Figure 20:**
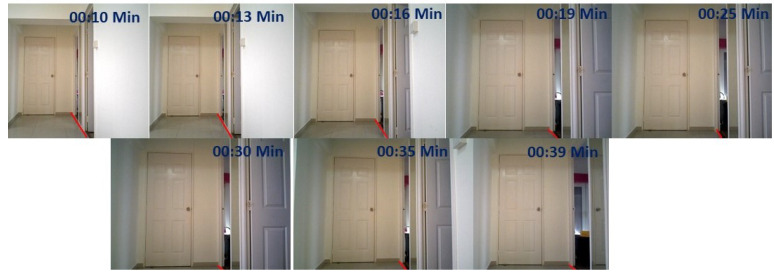
Series of image from the hsr robot during the trial with embedded time scenario 1.

**Figure 21:**
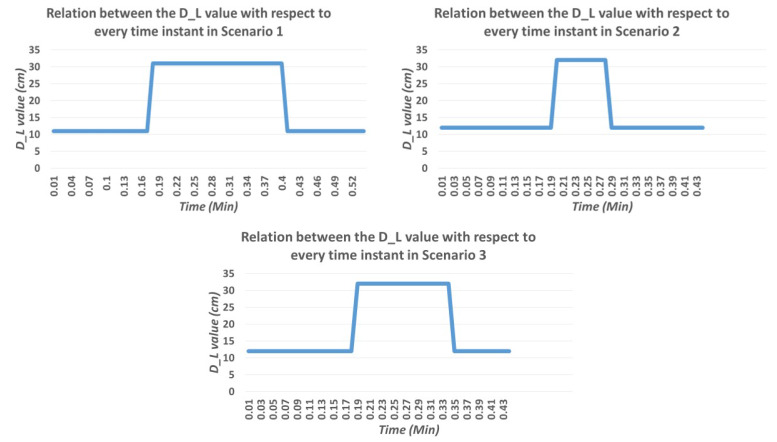
Time vs $D_{L}$ value curve in scenario 1 (**top left**), scenario 2 (**top right**), scenario 3 (**bottom**).

We evaluated the wall following the efficiency of the robot under the same set of scenarios. The wall following algorithm was running in the primary computing device. Once the wall/floor separation process is done the information of the separation line is passed to the primary device which runs the visual servoing scheme. The servoing scheme computes and generates the velocity commands with respect to the $D_{L}$ value. Since we are considering the proposed scheme for the wall cleaning purpose, the radius of the rotating cleaning brush is taken as a $D_{L}$. So in this case the $D_{L}$ value is taken as 20 cm. With the defined $D_{L}$ value, the robot follows the wall which is visualized in terms of path on the map as shown in Figure 19(top left). From the image, it is clear that the robot can successfully follow the wall. Figure 20(top left) shows the series of images taken from the robot’s camera embedded with timestamps of each frame. The door is actually detected at frame number four with time 00:19. The graph that depicts the time and $D_{L}$ relation in Figure 21(top left) shows the value increasing at the same time when the door is detected. That shows the accuracy of the robot to detect the obstacles and the ability to avoid the door. Figure 19(top left) also shows evidence of the avoidance ability of the robot at the end of the positions. There are few deviations in the path of wall following is due to the sensor noise that affects the localization and classification errors that happened in the primary computing device.

**Figure 22:**
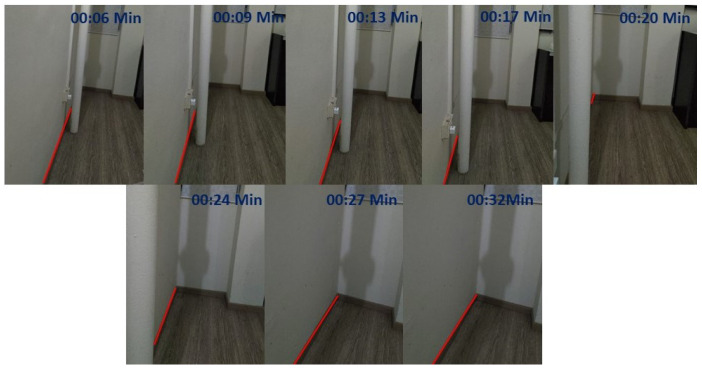
Series of image from the hsr robot during the trial with embedded time scenario 2.

In the second scenario, we conducted a similar trial with the HSR robot. Figure 19(top right) shows the localized position on the map, which is very near and mostly linear to the wall surface. Series of image frames with the timestamp is shown in Figure 22(top right) in which we can also witness the detection of PVC pipe at the time 0.20 min. Since the pipe is protruded out from a small distance, the $D_{L}$ for avoidance was increased in order to cross the obstacle. The graph shows the $D_{L}$ value with respect to the time of operation is shown in Figure 21(top right). From the above result, it has been proved that wall following and avoidance of the robot with the proposed system in scenario 2.

**Figure 23:**
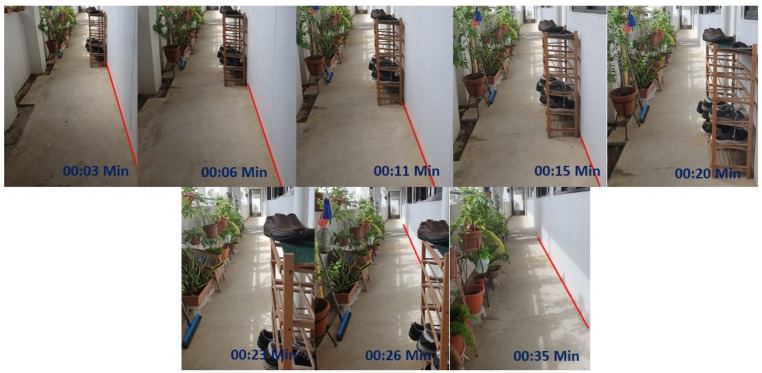
Series of image from the hsr robot during the trial with embedded time scenario 3.

Likewise, in scenario 3, the robot pursued the same procedure to execute the wall following task. In this trial, the robot is avoiding a shoe rack which is shown in one of the series of Figure 23 that is embedded on the wall side. Figure 19(bottom) shows the linear path with respect to the wall surface and gets diverted when it is near to the shoe rack. With regards to the $D_{L}$ value how the value has been changed with respect to the time is shown in Figure 21(bottom). From the results, it is clear that the proposed scheme could significantly improve the wall following efficiency with the HSR robot and can be extended to a similar mobile robot for various applications. Also, from the results, it is clear that the proposed methodology can definitely be applied for the wall cleaning task. Through this study, autonomous vision-based wall following system has been demonstrated in real-time that inaugurates a significant untapped research and development opportunity related to wall applications such as cleaning, painting, crack inspection, and so on.

## 5. Conclusions

In this article, we proposed a novel vision-based wall following framework that acts as an add-on for any professional robotic platform to perform wall only cleaning. The proposed scheme was constructed with two separate CNN networks, first is FCN8 which holds the liability of floor/wall segmentation process, and the second network is SSD mobile network that detects the obstacles embedded on the segmented wall surface and obstacle near by wall. We introduced visual servoing technique that uses the features extracted in the CNN layer to generate the motor commands that enables the robot to perform wall following task. Also, we explained the system architecture of the HSR robot which we are using as the experimental platform. We conducted experiments on three different scenarios to evaluate the proposed scheme with the HSR robot. The CNN networks are trained with various relevant data sets before we conduct the experiments. As per CNN concern, the system can significantly perform higher wall/floor segmentation rate and obstacle detection rate. The wall following results are shown as path tracings on the generated 2D map. The generated path tracing in all the experiments is mostly parallel to the wall which means the robot can successfully follow the wall surface in all considered scenarios. With respect to the obstacle avoidance, the path track shows the deviation from the wall surface at particular time instance from where the obstacle is detected. Overall the system shows significant performance in terms of wall-following tasks. Through this study, autonomous vision-based wall following system has been demonstrated in real-time that inaugurates a significant untapped research and development opportunity related to wall applications such as cleaning, painting, crack inspection, and so on.

There are few deviations in performance is due to the sensor noise that affects the localization and classification errors that happened in the primary computing device which will be eradicate through researching on following topics in the future works.

1. Improving the detection rate with training more images and different obstacles.
2. Extending the proposed framework to other wall following application such as painting, crack inspection.
3. Optimising the path generation of the robot to perform selective cleaning on wall.
4. Exploring on reinforcement learning techniques with the proposed scheme.

## Figures and Tables

**Table 1:** RGB-D camera specification.

Specification	Details
Dimensions	18×3.5×5
Resolution	SXGA (1280×1024)
Field of View	58${}^{\circ }$ H, 45${}^{\circ }$ V, 70${}^{\circ }$ D (Horizontal, Vertical, Diagonal)
Distance of Use	Between 0.8 m and 3.5 m
Power Consumption	Below 2.5 W
Data Rate	800 kb/s

**Table 2:** Performance measures-SSD MobileNet.

Class	Precision	Recall	F1	Accuracy	Confidence Level
Fire extinguisher	94.43	92.39	92.22	93.55	96
Sign board	91.24	90.31	90.03	90.19	92
Photo frame	90.45	90.09	89.96	90.11	94
wall ornaments	93.50	93.27	93.14	93.0	95
switch and safety box	92.75	92.33	91.89	92.32	92
Furniture	95.72	94.86	94.13	94.33	96
window and door	96.22	96.17	95.92	96.16	98
Dustbin and plant pot	93.65	93.34	92.93	93.44	96

**Table 3:** FCN8 Performance analysis.

Test Model	Pixel Accuracy (Average)	IOU	F1 Score
Offline Test	91.41	91.08	90.93
Real time with HSR	88.20	89.61	89.20

**Table 4:** Performance measures-SSD MobileNet.

Test Type	Precision	Recall	F1	Accuracy
Offline (average result)	93.45	92.84	92.52	92.88
Real-time (average result)	90.3	88.7	89.5	86.0

**Table 5:** Segmentation framework comparison analysis.

Algorithm	Pixel-Accuracy	IOU	F1 Score
FCN-AlexNet	83.13	81.30	81.8
FCN16	89.64	89.79	89.88
FCN8	91.41	91.08	90.93

**Table 6:** Comparison with other object detection framework.

Test	Faster RCNN ResNet				Faster RCNN Inception				Yolo v2			
	Prec.	Recall	$F_{1}$	Accuracy	Prec.	Recall	$F_{1}$	Accuracy	Prec.	Recall	$F_{1}$	Accuracy
Fire extinguisher	97.33	96.91	96.84	96.93	95.12	94.89	94.55	94.07	89.22	88.09	88.02	89.07
Sign board	96.50	96.14	96.05	96.28	93.92	93.50	93.33	93.37	86.71	86.18	85.90	86.08
Photo frame	96.30	95.79	95.75	95.98	92.79	92.63	92.31	92.30	86.39	85.13	85.05	85.66
wall ornaments	97.72	97.47	97.17	97.18	94.35	94.26	94.19	94.12	88.89	88.65	88.46	88.55
switch and safety box	93.98	93.23	92.94	93.43	91.99	91.78	91.76	91.80	84.90	84.49	84.31	84.46
Furniture	97.85	97.80	97.72	97.40	96.82	96.56	96.37	96.57	90.70	90.12	89.97	90.03
window and door	97.25	97.18	97.11	97.09	96.29	95.92	95.86	96.04	89.53	89.27	89.16	89.28
Dustbin and plant pot	96.60	96.32	96.28	96.39	95.79	95.27	95.25	95.20	89.86	89.45	89.33	89.40
